# Optical and Mechanical Properties of Highly Translucent Dental Zirconia

**DOI:** 10.3390/ma13153395

**Published:** 2020-07-31

**Authors:** Hee-Kyung Kim

**Affiliations:** Department of Prosthodontics, Institute of Oral Health Science, Ajou University School of Medicine, Suwon 16499, Korea; denthk@ajou.ac.kr

**Keywords:** zirconium oxide, lithium disilicate, translucency, opalescence, fluorescence

## Abstract

The aim was to evaluate the translucency, opalescence, and fluorescence of highly translucent zirconia, lithium disilicate, and bovine teeth. One mm-thick specimens of five monolithic zirconia systems, two glass-ceramics, and bovine enamel/dentin were investigated. A spectrophotometer (Ci7600) was used to measure the CIELab color coordinates, and the translucency and opalescence values were obtained. For evaluating the fluorescence emission, the differences in spectral reflectance by the UV component of illumination were calculated. The microstructures of ceramic specimens were examined with a scanning electron microscope and the chemical compositions were determined with an X-ray fluorescence spectrometer. Mechanical properties were appraised with three-point bending strength, indentation fracture toughness, and Vickers hardness. Data were analyzed using a one-way ANOVA, followed by Tukey’s multiple comparison test (α = 0.05). A higher yttria content (5 mol%) significantly improved the translucency of zirconia ceramics, while they were less translucent than lithium disilicate (*p* < 0.05). Lowering the alumina content below 0.05 wt.% enhanced the translucency (*p* < 0.05), but a small amount of alumina was still required to obtain full densification. 0.05 wt.% Fe was used to increase the chroma of zirconia specimens without compromising their mechanical properties. The Er-containing zirconia specimen showed a maximal fluorescence emission at 430 nm. The degree of opalescence was affected by the microstructures of ceramic materials. The microstructure, incorporation of a secondary phase, and sintering behavior can have a strong impact on the final mechanical and optical properties of dental ceramics. Addition of small amounts of metal oxides can affect the translucency, opalescence or fluorescence qualities of zirconia

## 1. Introduction

Zirconia has become widely used in dentistry for restorations and implants due to its high strength and inherent whitish color [1]. It can be manufactured in a wide variety of shades, making it easy to accurately match to the color of the patient’s natural teeth. However, its opaque appearance caused some esthetic failures and thus, further improvements of the optical properties have been made.

Zirconia is a polymorphic material occurring in three temperature-dependent forms: monoclinic (stable at room temperature), tetragonal (at about 1173 °C), and cubic (at about 2370 °C) [2]. In an attempt to improve the translucency of standard 3 mol% yttria partially stabilized tetragonal zirconia polycrystal (3Y-TZP) grades, cubic-phase crystal can be stabilized at room temperature by increasing yttria contents (4–5 mol%); 4 mol% yttria partially stabilized zirconia (4Y-PSZ) or 5 mol% yttria partially stabilized zirconia (5Y-PSZ) [1]. Those highly translucent dental zirconia have received a great deal of attention in recent years, due to their enhanced light transmittance, and as a result, anatomic-contour zirconia in the monolithic form has expanded the range of indications, allowing its use in the anterior esthetic zone [3]. The structure and grain size of the cubic crystal improved the optical physics of zirconia. The crystal of the cubic system is optically isotropic and thus, the refractive index is equal in all directions throughout the crystal lattice. Furthermore, the cubic polymorph of zirconia has a grain size beyond the red portion of the visible wavelengths resulting in less grain-boundary area per unit volume. Those above-mentioned optical properties would cause less light scattering at the grain boundary, contributing to the greater amount of light transmittance significantly [4]. The introduction of new cubic varieties of zirconia markedly enhanced translucency [5,6,7], but the mechanical strength was reduced compromising its clinical use in long-span posterior bridges [6,8,9].

As the demand for esthetics increases in dental practice, the reproduction of the lifelike opalescence and fluorescence as well as translucency is required to mimic the appearance of a natural tooth. The opalescence effect in tooth enamel can be produced by the scattering of the visible light. A natural tooth appears to be bluish under reflected light and orange-brown under transmitted light [10], because the shorter wavelength (blue light) would scatter more than the longer wavelength (yellow or red light) according to the effect of Rayleigh scattering [11]. Opalescence which can increase the lightness of the tooth and provide optical depth and vitality [12], was considered as a determining factor for the color of the natural tooth, along with hue, value, chroma, and characterization [13].

Light scattering is caused by the refractive index mismatch between the internal particle and the matrix. The internal particle in a size of 450-nm wavelength blue light can induce the scattering of short-wavelength visible light, allowing blue light to be reflected [14]. Egen et al. reported that the material could exhibit opalescence when the refractive index ratio between the phases was 1.1 or more [15]. Hydroxyapatite crystals in tooth enamel play a considerable role in the light scattering process, contributing to the opalescent effect [16]. For dental restorative ceramic materials, although the microstructure and the compositions varied, light scattering by finely-dispersed small internal particles (380–500 nm) can cause opalescence [14]. The opalescence values of resin composites or core/veneer ceramics have been investigated. The values were varied by the kind of materials or the presence of particles, but the values were lower than that of tooth enamel [17,18].

A natural tooth can fluoresce a blue-white hue. Fluorescence is the optical property, whereby a tooth absorbs ultraviolet (UV) light and then the activated electron returns to a lower energy level, making it emit blue light (440–490 nm) in the visible light spectrum [19,20]. Tooth dentin was found to be more fluorescent than enamel due to photosensitive organic components (fluorophores) [21,22]. In addition to the tooth compositions, its structural features, such as dentinal tubules, secondary dentin, inter-globular spaces, enamel lamellae, or Hunter-Schreger bands in tooth enamel, could increase the intensity of fluorescence [21]. It was also reported that the intensity of tooth fluorescence increased with age, while it was not related to tooth types [19]. The fluorescent restorative material can offer the lifelike-quality and is especially beneficial for the dark tooth as it can increase value without adversely affecting the translucency [10].

The fluorescent characteristics of rare earth elements have been investigated. The pure salts of the rare earths excited by ultraviolet light exhibited fluorescent brightness [23,24]. Jenkins and McKeag demonstrated that some phosphors activated by rare earths contributed to the fluorescence emission [25]. Dental restorative materials are generally lacking a fluorescent property, and, therefore, small amounts of rare earths such as ytterbium, cerium, terbium, europium, or thulium can be added as luminophores for dental porcelain [26], composite resin [27], and zirconia [28,29] to simulate natural fluorescence. Additionally, zirconia has been tested recently concerning its mechanical properties. The bond strength [30], hardness [31], and wear [32] tests have been performed on this material, showing excellent mechanical properties.

To ensure optimal esthetics, an understanding of the optical properties of natural teeth is important. Due to the difficulty in obtaining human teeth and a limitation to get flat surfaces of uniform thickness, bovine teeth have been used as substitutes for human teeth in dental research studies. Although there can be some differences in the structure and chemistry between human and bovine teeth [33], it was reported that human dentin and bovine dentin exhibited similar spectral reflectance characteristics [34]. In the study of Yu et al., human enamel/dentin showed higher translucency values than those of bovine enamel/dentin [35].

Although previous studies evaluated the optical and mechanical properties of dental ceramics, very little information is available about the translucency, opalescence, fluorescence, and mechanical properties of new highly translucent cubic zirconia. The purpose of the present study was to investigate the translucency, opalescence, and fluorescence of highly translucent zirconia, lithium disilicate, and bovine teeth. Moreover, this study intended to investigate the effect of the microstructures and specific metal oxide additives on the optical and mechanical properties of dental ceramics. In this study, careful separation of the enamel-dentin unit was made by the grinding and polishing of enamel layers to expose dentin surfaces. The null hypothesis tested was that the translucency, opalescence, and fluorescence values of highly translucent cubic zirconia would not differ from those of 3Y-TZP, lithium disilicate, and bovine teeth.

## 2. Materials and Methods

Enamel and dentin specimens as reference groups were prepared from 20 extracted bovine incisors. The teeth without visible caries or cracks, and visually matched to A2 shade of VITA classical shade system (VITA Zahnfabrik, Bad Säckingen, Germany) were selected. The 10 enamel disc specimens (10 mm in diameter) were obtained from the labial surface with a trephine mill (Komet Brasseler GmbH & Co., Lemgo, Germany) [36]. For the other 10 crowns, labial surfaces were ground with a 600-grit carborundum disc under a stream of running water to remove enamel layers and then 10 dentin disc specimens (10 mm in diameter) were acquired using a trephine mill. The specimens were ground flat and were polished with a 1000-grit silicon carbide paper in a lapping and polishing unit (Labopol-5, Struers, Copenhagen, Denmark) with water irrigation at a low 150 rpm until obtaining 1.0 mm thick slices. The thicknesses were verified to a precision of 0.01 mm with a digital micrometer (Digimicro ME-50HA, Nikon Corp., Tokyo, Japan). Afterwards, the specimens were stored in 0.1% thymol solution before measurements to prevent dehydration [37].

Three commercially available 5Y-PSZ pre-colored monolithic zirconia materials (Lava Esthetic, LET/LEB (3M ESPE, St. Paul, MN, USA); BruxZir Anterior, BA (Glidewell, Newport Beach, CA, USA); and Luxen Smile, SMS2 (; DENTALMAX, Seoul, Korea) were cut and polished (both sides) to a mirror-like finish (1μm) by using a lapping and polishing machine (SPL-15 GRIND-X; Okamoto, Japan). For Lava Esthetic, which is a multilayer shaded 5Y-PSZ material, the top surface (LET) and the bottom surface (LEB) were selected for the measurement of the optical properties. The 3Y-TZP materials used were Lava Plus (LP, colorless) and Luxen Zr (S2, pre-colored) as controls. For the measurements of the translucency, opalescence, and fluorescence, the final dimensions of the zirconia specimens were 15 mm × 15 mm × 1 mm (*n* = 10 for each zirconia group). For the flexural strength, the final dimensions of the zirconia specimens were 4 mm × 3 mm × 40 mm (*n* = 10) and 15 mm × 15 mm × 5 mm (*n* = 5) for the fracture toughness testing. The lithium disilicate materials selected were e.max CAD (computer-aided design) HT (high translucent) and LT (low translucent) as references. The specimens were sectioned and underwent a crystallization process following the manufacturer’s instructions; 14 mm × 14 mm × 1 mm (*n* = 10 for each e.max group), 4 mm × 3 mm × 33 mm (*n* = 10 for each e.max group), and 14 mm × 14 mm × 5 mm (*n* = 5 for each e.max group). All ceramic specimens used in the present study were selected corresponding to the A2 shade in the VITA classical shade guide, except Lava Plus (white). The characteristics of the ceramic systems investigated are provided in Table 1.

Spectral data were recorded from 360 to 750 nm at 10 nm intervals in the reflectance mode against a white, a black, and A2 ceramic background in UV-included and UV-excluded modes, and in the transmittance mode on a reflection spectrophotometer (Ci7600; X-Rite, Grand Rapids, MI, USA). For the reflectance measurement, CIE diffuse/8° illuminating/viewing configuration with a 6 mm diameter aperture and a 6 mm diameter measurement area was used. For the transmittance measurement, CIE diffuse/0° illuminating/viewing configuration with a 6 mm diameter aperture and a 6 mm diameter measurement area was used. The CIELab color coordinates were calculated from the spectral distributions with the 2° standard observer and standard illuminant D65: The L* value represents the brightness of an object, the a* value represents the red or green chroma, and the b* value represents the yellow or blue chroma [38]. The measurements were conducted by using glycerin as an optical coupling medium in-between the specimen and the background [39]. Three measurements were performed on each specimen, and the average value was then calculated.

The translucency parameter (TP) value was determined by calculating the CIEDE2000 color differences ($∆E_{00}$) between the values against a white and a black background [38,40] as

$$∆E_{00}={\left[{\left(\frac{∆L{}^{\prime }}{K_{L}S_{L}}\right)}^{2}+{\left(\frac{∆C{}^{\prime }}{K_{C}S_{C}}\right)}^{2}+{\left(\frac{∆H{}^{\prime }}{K_{H}S_{H}}\right)}^{2}+{\;R}_{T}\left(\frac{∆C{}^{\prime }}{K_{C}S_{C}}\right)\left(\frac{∆H{}^{\prime }}{K_{H}S_{H}}\right)\right]}^{\frac{1}{2}}$$

where $∆C{}^{\prime }$ and $∆H{}^{\prime }$ are the differences in chroma and hue for a pair of specimens. The total transmittance (T%) was calculated [41] as: T% = $\left(\frac{L^{*}{}_{\mathrm{specimen}}}{L^{*}{}_{\mathrm{source}}}\right)$ × 100, where L^*^_source_ was obtained without any specimen placed before each measurement. The L^*^_specimen_ was recorded from 360 to 750 nm and the mean T% values at 550 nm wavelength were used for the comparison [42].

The opalescence parameter (OP) value was obtained by calculating the CIEDE2000 color differences ($∆E_{00}$) between the reflected color against a black background and the transmitted color [14]. For evaluating fluorescence emission, the differences between the spectral reflectance under UV-included condition and that under UV-excluded condition at each wavelength from 400 to 750 nm were calculated [43].

One specimen from each group was imaged with a scanning electron microscope (SEM; JSM-7900F; JEOL Ltd., Tokyo, Japan) and the images of each specimen were obtained at magnifications of 5000×, 10,000×, and 30,000×. The zirconia specimens were thermally etched in air at 1350 °C for 30 min with fast heating rate (20 °C/min) and the lithium disilicate specimens were prepared with 5% hydrofluoric acid (IPS Ceramic Etching Gel, Ivoclar Vivadent AG, Schaan, Liechtenstein) for 20 s. Subsequently, each specimen was cleaned with 95% alcohol and the specimen surface was coated with a thin layer of platinum (108 Auto Sputter Coater; Cressington, Watford, UK). The SEM analysis was conducted in a vacuum (9.6 × 10^−5^ Pa) at an acceleration voltage of 15.0 kV and an emission current of 64.2 µA. The grain sizes were measured at 10 different locations on each specimen.

The chemical compositions of ceramic specimens (*n* = 3) were determined by a wavelength dispersive X-ray fluorescence spectrometer (WD-XRF; ZSX Primus; Rigaku, Tokyo, Japan) using a rhodium (Rh) tube, an anode voltage up to 60 kV, and an electric current up to 150 mA. The pressed specimens were measured in a vacuum atmosphere. Measurements were performed using a ZSX Guidance software (v3.34).

The mechanical properties were measured according to ISO 6872:2015 [44]. The hardness and fracture toughness were obtained by the Indentation Fracture (IF) method [45] on polished specimen surfaces by using a Vickers micro-hardness tester (Z2.5; Zwick/Roell, Ulm, Germany) with a load of 10 kg for 10 s. Five indentations were made at each load for each specimen.

Flexural strength was determined by using 3-point bending (span length of 30 mm) of bar specimens in a universal test machine (RB 301 UNITECH-M; R&B, Daejeon, Korea) with a 5 kN load cell (UM-K500; DACELL, Cheongju, Korea), at a cross head speed of 1 mm/min until failure occurred [46].

The color values, translucency, opalescence, and mechanical properties of different materials were compared using the one-way analysis of variance (ANOVA), followed by Tukey’s multiple comparison test. The expected statistical powers with the chosen sample size were 95.1% for the optical properties and 89.1% for the mechanical properties to detect specified differences (G*Power v3.1.9.2; Duesseldorf University, Dusseldorf, Germany). For all of the analyses, the statistical software (IBM SPSS Statistics for Windows, v23.0, IBM Corp., Chicago, IL, USA) was used. The statistically significant level was set at 0.05.

## 3. Results

**Figure 1:**
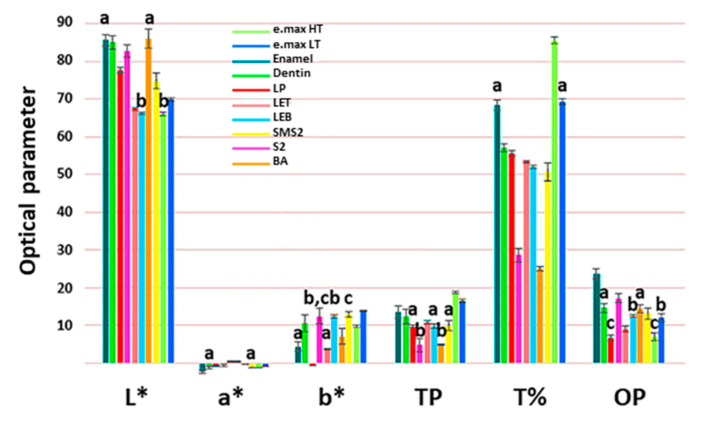
Optical parameters of CIEL*, a*, and b* against an A2 background, TP, T% at 550 nm, and opalescence parameter (OP) of each group. Means with the same superscript letter in each column are not significantly different from each other based on Tukey’s multiple comparison test (*p* > 0.05).

**Figure 2:**
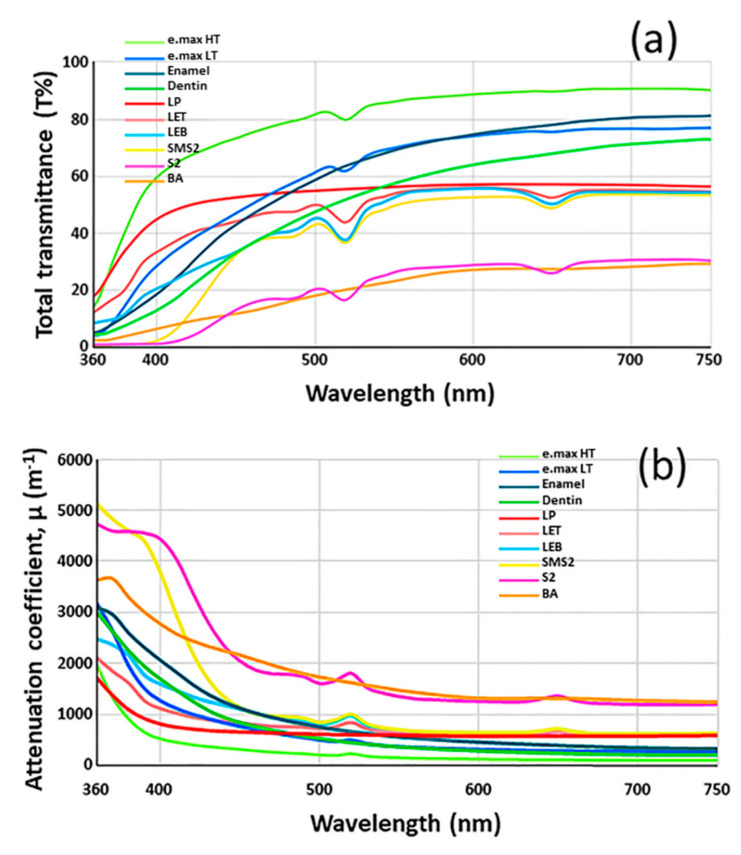
(**a**) Spectral light transmittance (T%) of each group as a function of wavelength. The e.max HT exhibited the highest transmittance value. (**b**) Attenuation coefficients (μ) as a function of wavelength. The attenuation coefficients decreased as the wavelength increased.

The CIELab color values demonstrated a normal distribution according to the Shapiro-Wilk test (*p* < 0.05). The CIELab color values against an A2 background, TP, T% at 550 nm, and OP values of each group are visualized in Figure 1. The highest TP value was found in e.max HT, while the lowest TP value was observed in S2. The highest OP value was found in bovine enamel, while the lowest OP value was observed in LP. The OP value of S2 was higher than that of bovine dentin. Spectral light transmittances (T%) of each group as a function of the wavelength are shown in Figure 2a. The attenuation coefficients (μ) as a function of the wavelength were also determined [47], which are shown in Figure 2b. The attenuation coefficients decreased as the wavelength increased. The e.max HT exhibited the highest transmittance value as high as 75% above 500 nm, while 3Y-TZP specimens showed the lowest transmittance in the visible light region. Moreover, the transmittances of 5Y-PSZ groups were higher than those of 3Y-TZP groups. The transmittance of the bovine enamel was significantly higher than that of bovine dentin.

**Figure 3:**
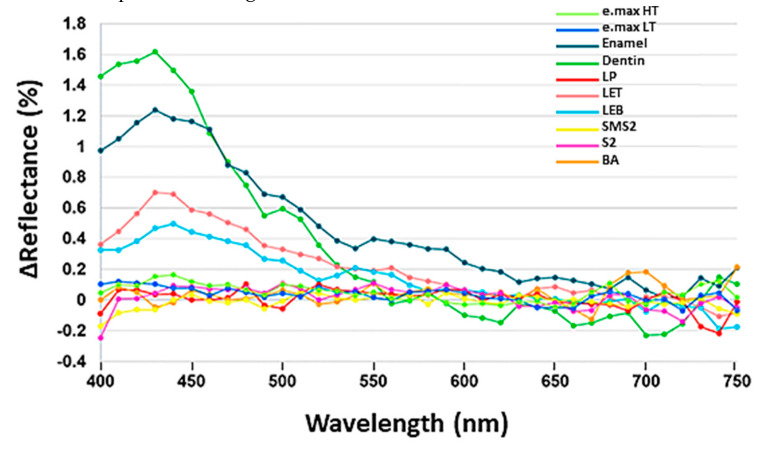
Differences in the reflectance under different UV illumination conditions. Of ten specimens, four showed fluorescence.

Differences in the reflectance under different UV illumination conditions are shown in Figure 3. Bovine dentin exhibited highest fluorescence value. Bovine dentin, enamel, LET, and LEB showed fluorescence and the peak wavelength was 430 nm.

**Figure 4:**
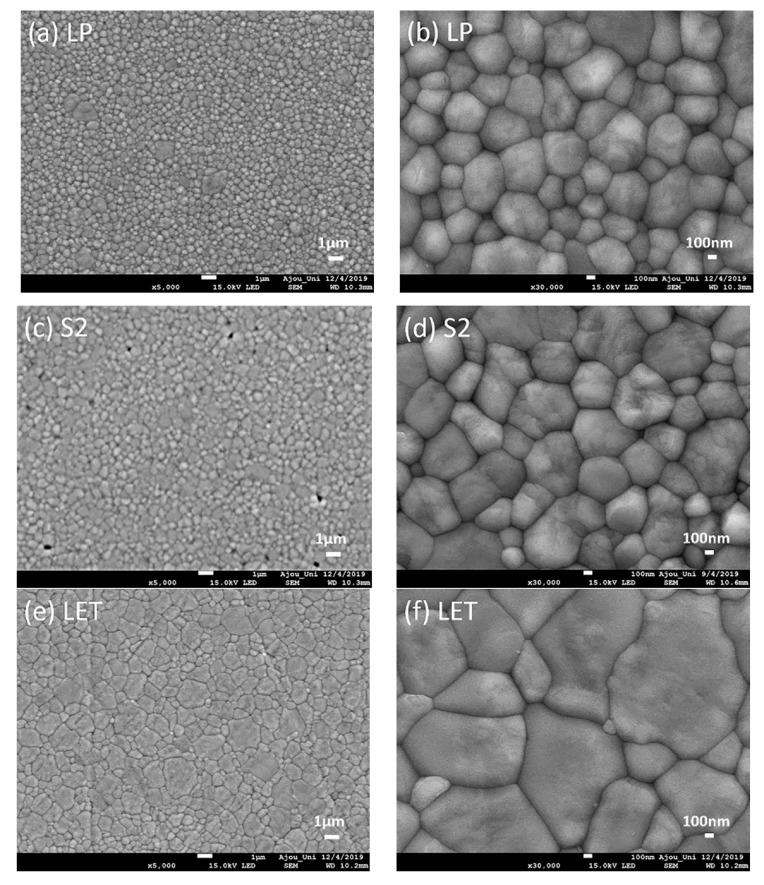

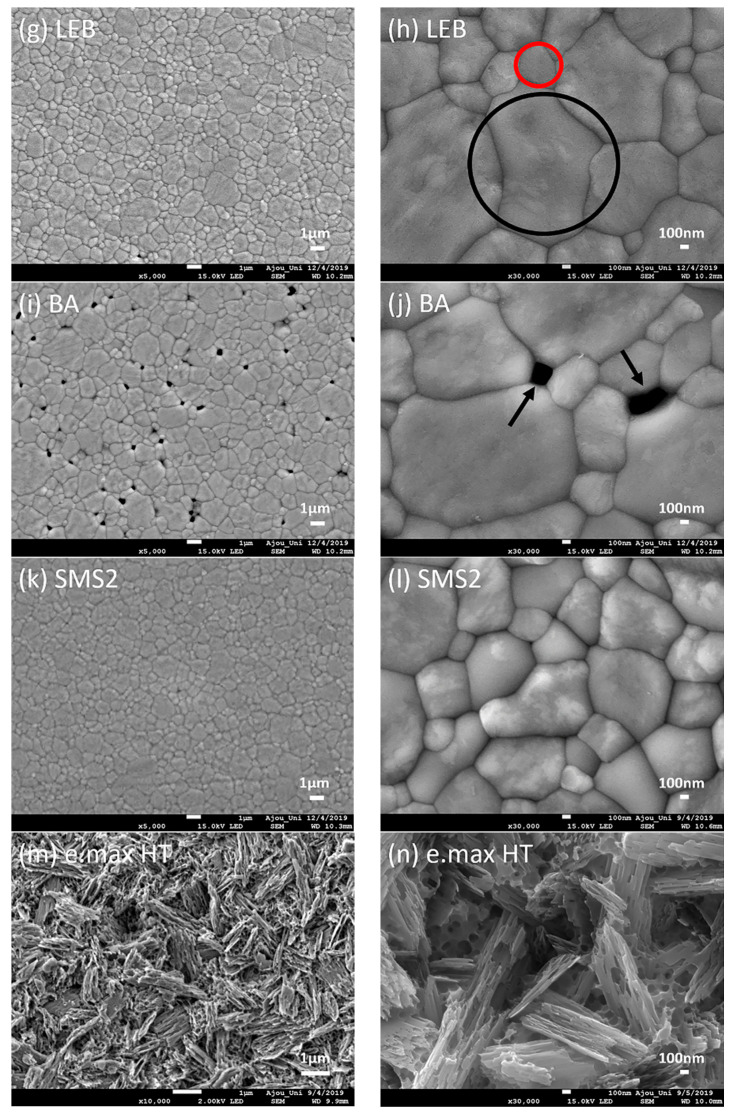

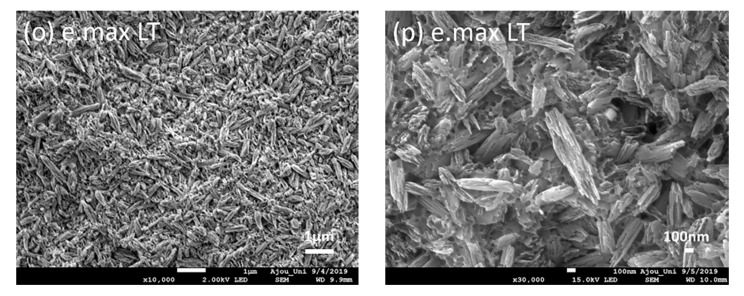
SEM images of ceramic specimens. 3Y-TZP: (**a**) LP (5000×), (**b**) LP (30,000×), (**c**) S2 (5000×), (**d**) S2 (30,000×); 5Y-PSZ: (**e**) LET (5000×), (**f**) LET (30,000×), (**g**) LEB (5000×), (**h**) LEB (30,000×), (**i**) BA (5000×), (**j**) BA (30,000×) (**k**) SMS2 (5000×), (**l**) SMS2 (30,000×); Glass-ceramic: (**m**) e.max HT (10,000×), (**n**) e.max HT (30,000×), (**o**) e.max LT (10,000×), (**p**) e.max LT (30,000×). The micrographs of 3Y-TZP specimens depicted grain sizes that approached nanometric scales while 5Y-TZP specimens presented bigger grain sizes, approaching to micrometric scales. The black arrow (**j**) indicates the pore; the red circle (**h**) indicates a tetragonal grain; the black circle (**h**) indicates a cubic grain of zirconia.

Representative SEM images of the ceramic specimens were shown in Figure 4 (3Y-TZP: Figure 4a–d; 5Y-TZP: Figure 4e–l; glass-ceramic: Figure 4m–p). The average grain size of LP was the smallest (approximately 344 nm, Figure 4a,b). The 3Y-TZP specimens had uniformly dispersed sub-micrometric tetragonal grains (approximately 280–360 nm), whereas the bimodal grain size distributions were found for 5Y-PSZ specimens (approximately 543–1680 nm), with larger cubic grains (≥1.2 µm) embedded in the tetragonal grain structures. The pores or voids were visible at the grain interfaces in BA (Figure 4i,j). The microstructures of glass-ceramics consisted of high content (~70 vol%) of elongated lithium disilicate crystals embedded in a glassy matrix. The average grain size of e.max HT was 150–450 nm in width and 1–3 µm in length (Figure 4m,n), while e.max LT had smaller lithium disilicate crystals with higher crystalline content (Figure 4o,p).

According to XRF analysis, the erbium (Er) rare-earth metal was identified in LET, LEB, and SMS2. As trace elements, aluminum (Al), iron (Fe), and calcium (Ca) ions were detected for zirconia. Al was detected in LP (0.06 wt.%), S2 (0.05 wt.%), LET/LEB (0.03 wt.%), and SMS2 (0.02 wt.%). Fe was found in S2 (0.05 wt.%) and SMS2 (0.05 wt.%). Ca was detected in BA (0.02%). The e.max LT had higher Al content than that of e.max HT. The chemical compositions of materials determined by XRF are listed in Table 2.

**Figure 5:**
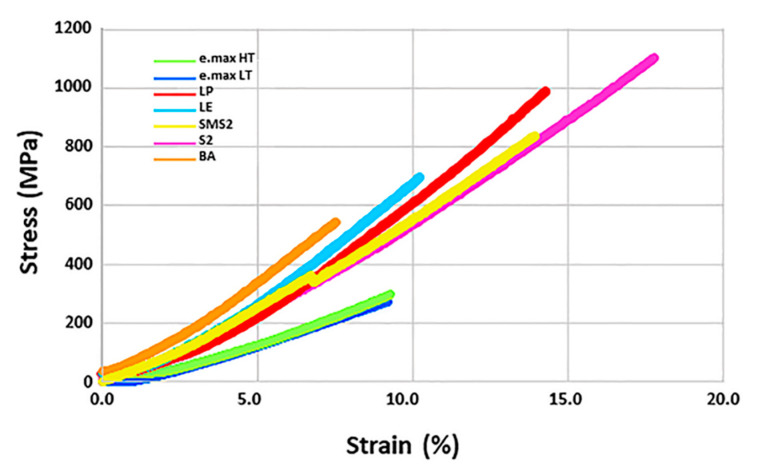
Stress-strain curves. S2 showed the highest ductility.

The physical and mechanical properties of the ceramic specimens investigated are shown in Table 3. Glass-ceramics demonstrated the lowest flexural strength and fracture toughness compared with the zirconia specimens (*p* < 0.001). For the zirconia materials, 3Y-TZP specimens showed higher flexural strength and fracture toughness values than those of 5Y-PSZ specimens (*p* < 0.001). With respect to density, the lowest value (5.819 g/cm^3^) was found for BA among zirconia specimens. Stress-strain curves of the ceramic specimens are illustrated in Figure 5, indicating zirconia groups have higher yield stresses than e.max groups. Furthermore, S2 showed the highest ductility [48].

## 4. Discussion

The present study evaluated the translucency, opalescence, and fluorescence of highly translucent zirconia, lithium disilicate, and bovine teeth. The results showed that the optical properties varied depending on the type of ceramic specimens. Thus, the null hypothesis, that the translucency, opalescence, and fluorescence values of highly translucent cubic zirconia would not differ from those of 3Y-TZP, lithium disilicate, bovine teeth, was rejected.

Several studies investigated the translucency of new highly translucent cubic zirconia in comparison with 3Y-TZP or glass-ceramics. Camposilvan et al. [5] reported that the translucency improved with increasing the cubic phase content, but the highly translucent cubic zirconia still has limitations for use in the anterior region. Carrabba et al. [6] showed that the translucency of 5Y-PSZ lay between 3Y-TZP and lithium disilicate glass-ceramic. On the contrary, cubic zirconia exhibited higher translucency than that of lithium disilicate glass-ceramic providing restorations of high esthetic outcome in the study of Baldissara et al. [7]. This study used TP and T% values to evaluate the material translucency. The e.max HT showed the highest translucency, even higher than that of bovine enamel. Currently, e.max CAD is available in three different degrees of translucency: MO (moderate opaque), LT (low translucent), and HT (high translucent). As seen in the SEM images in this study, HT showed larger size crystallites with a lower crystal density compared to LT, leading to less light scattering. XRF analysis indicated that e.max HT had Zn (3.98%) and Sr (0.06%) which were not in e.max LT. The lithium disilicate is known to undergo a crystallization process to precipitate lithium disilicate and the initial glass composition could affect lithium disilicate crystallization. The incorporation of some components such as La_2_O_3_, MgO, ZnO, B_2_O_3_, and Al_2_O_3_, can modify lithium disilicate composition and favor the crystallization [49]. Based on the result of this study, Zn which was not present in e.max LT, might play a crucial role in crystal growth in e.max HT. Moreover, the refractive index of the lithium disilicate (~1.55) is close to that of matrix glass (~1.5), which thus enabled the creation of glass-ceramics with high translucency. Specifically, the refractive index of the glass matrix can be increased to match that of the lithium disilicate phase by doping small amounts of heavy metal oxides such as Sr, Y, Nb, Cs, Ba, Ta, Ce, or Eu oxides [49]. In this study, small addition of Sr to e.max HT might contribute to high translucency by reducing the refractive index mismatch.

For 3Y-TZP materials used in this study, LP was a non-colored white zirconia material, while S2 was a pre-colored zirconia material. It was reported [50] that the addition of Fe_2_O_3_ into the zirconia matrix could give it beige chroma without sacrificing its mechanical properties. As shown in the spectral and XRF analyses in this study, Fe could be an essential element responsible for creating color by an increase in b^*^ value. Furthermore, S2 showed higher strength and toughness values than those of LP. It can be assumed that Fe_2_O_3_ doping as a coloring element into the zirconia matrix might not adversely affect its mechanical strengths.

The conventional dental zirconia (3Y-TZP) contained 0.25 wt.% alumina (Al_2_O_3_) sintering aid, and the positive effect of an alumina addition on the mechanical properties and degradation resistance of Y-TZP ceramics has been widely investigated [2,51]. Zhang et al. [51] reported that the optimal alumina quantity for the aging resistance effect was related to the solubility of alumina in zirconia. Recently, attempts have been made to increase the translucency of zirconia by decreasing the amount of alumina dopant to 0.1 or 0.05 wt.%, since the refractive index mismatch between alumina (*n* = 1.765) and zirconia (*n* = 2.175) can cause significant light scattering although its hydrothermal stability was sacrificed [2]. Based on the XRF results of this study, all monolithic zirconia specimens (3Y-TZP and 5Y-PSZ) except BA contained less than 0.06 wt.% alumina (Al_2_O_3_), and BA specimens even eliminated the alumina contents. All zirconia specimens in this study had 1.77–2.76 wt.% Hf. Zirconium (Zr) and hafnium (Hf) are normally associated in nature and thus, it is difficult for them to be separated due to their similar chemical structures [52]. However, the strong neutron-absorption effect of Hf would be harmful for Zr [52].

For the 3Y-TZP zirconia specimens in this study, LP exhibited higher translucency values (TP and T%) than those of S2. The coloring pigment of S2 could negatively affect its translucency. In addition, relatively finer grain sizes of the LP specimen would contribute to improve the translucency by increasing the in-line transmission [2]. The translucency of 5Y-PSZ specimens in this study substantially increased due to the introduction of an optically isotropic cubic zirconia phase with a higher yttria content. As seen in the SEM images in this study, larger grains with the reduced grain-boundary light scatterings can account for the enhanced translucency in 5Y-PSZ materials, although the translucency values of 5Y-PSZ specimens did not reach that of bovine tooth specimens. However, the strength and toughness decreased because cubic zirconia crystal does not exhibit stress-induced transformation toughening [2].

In this study, Ca was used as a coloring agent for BA [53]. Contrary to expectation, the results revealed that alumina-free BA, where the highest yttria concentration (9.7067 wt.%) was utilized, exhibited low value of translucency, even lower than those of 3Y-TZPspecimens. This may be due to the residual pores at the grain boundaries which was seen in the SEM image since the presence of pores could be possible sources of the light scattering [5]. It was reported that the dopant played a significant role in the densification behavior of ceramics by controlling the grain boundary mobility [53]. Higher sintering temperature was necessary to achieve full densification for Y-TZP with small alumina contents (0.01 wt.%) [54]. Moreover, even minor alumina addition could play an important role on the aging resistance of Y-TZP [55]. Zhang et al. [4] investigated that alumina-free 3Y-TZP did not exhibit higher translucency than that of 3Y-0.05Al, and the alumina-free 3Y-TZP had weaker grain boundary than that of alumina-doped 3Y-TZP yielding the inter-granular fractured surfaces. Therefore, different sintering processes (temperature and time) might be needed to accomplish full densification of alumina-free BA.

The range of opalescence parameter was 6.38–22.62 for the materials used in this study. Among them, bovine enamel showed the highest value. For ceramic specimens, the highest opalescence was observed in S2. As previously discussed, the presence of particles whose size is in the visible wavelength range and a large difference in the refractive index between phases could contribute to opalescence [14]. For S2, finely dispersed zirconia grains (approximately 430–616 nm) would cause opalescence. In addition, the refractive index mismatches between some oxides, such as: ZrO_2_ (2.175), Y_2_O_3_ (2.1585), Fe_2_O_3_ (2.918), and Al_2_O_3_ (1.765), could increase light scattering. Shiraishi et al. [56] demonstrated that the opalescence of dental ceramics increased with increasing chroma or value due to the increased concentrations of metal oxides. As opposed to S2, LP demonstrated weakest opalescence because its small grain sizes (between about 300 and 347 nm) might be less effective in creating opalescence. The glass ceramics in this study showed some opalescence, but it was reported that the columnar crystals in the dental ceramic induced less light scattering than the sphere crystals [14]. Furthermore, zirconia specimens in this study generally exhibited opalescence and their microstructures could affect opalescence. For BA, the pore between grain boundaries would cause opalescence.

The addition of small amounts of rare-earth elements to zirconia can provide the fluorescence emission under UV lighting to mimic that of natural teeth [29]. The Er_2_O_3_ can be added as a fluorescent pigment as well as a coloring agent [24,57]. However, the preferred range of Er as Er_2_O_3_ would be 0.0001–0.5 wt.% [57]. As illustrated in Figure 3 in the present study, LET and LEB which contained trace amounts of Er showed fluorescence when exposed to UV light. However, SMS2 with 1.0339 wt.% of Er did not exhibit fluorescence. The peak fluorescent intensity would vary depending on the amount of the pigment due to the concentration Quenching [58].

The reproduction of the optical properties of natural teeth can be designed by controlling the translucency, opalescence, and fluorescence of ceramic materials. The compositions, the size and volume fractions of additives, and the microstructural features of dental ceramics can govern the light physics. Although there have been a number of studies on the translucency of dental esthetic materials, there are few studies on the opalescence and fluorescence of high-performance dental ceramics. In this study, it was found that cubic grains of highly translucent zirconia contributed to enhance the translucency of zirconia, but its translucency did not reach that of lithium disilicate or bovine teeth. In addition, the pores between the zirconia grains and the secondary phase of component had a negative effect on the translucency. The microstructure of ceramic materials could be an important factor for controlling the translucency and opalescence. The fluorescence could be created by incorporating rare-earth ions into a ceramic matrix.

The application of CAD/CAM technology in dentistry has led to the increasing adoption of zirconia with improved esthetics. CAD/CAM systems registered a constantly increasing use in many fields of dentistry, such as restorative dentistry, prosthodontics, and orthodontics. CAD/CAM technology allows a completely digital workflow, from impression to final framework, with clinical reliability [59] and good patient’s feedback [60]. Therefore, CAD/CAM systems are essential to the continued success of zirconia as an esthetic dental material.

One of the limitations of this study is that bovine teeth were used as a substitute for human teeth due to the difficulty in obtaining specimens of the correct size with uniform thickness. Although it has been reported that the reflectance spectral behavior of bovine teeth and human teeth were similar [34,35], direct application of data obtained from bovine to human teeth may not be considered because their chemistry and structure are not identical [33]. Therefore, the results presented in this study suggest that used bovine teeth are not necessarily fully applicable to human teeth. Human teeth substrates should be used as a positive control for further studies on the optical physics of dental ceramics due to their clinical relevance. Another limitation was the use of a spectrophotometer with a small window for spectral measurements, which could result in the edge loss effects [31]. Accordingly, optical distortions would be inevitable.

## 5. Conclusions

Within the limitations of this study, the results indicated that, highly translucent zirconia has a significantly higher translucency than 3Y-TZP but is not as translucent as lithium disilicate or bovine teeth. Dental ceramics are less opalescent than bovine enamel. The incorporation of small amounts of rare-earth ions into ceramic systems can control the fluorescence quality. The crystal growth kinetics of lithium disilicate could lead to enhanced translucency. Therefore, the microstructure, the incorporation of a secondary phase, and the sintering behavior can have a strong impact on the final mechanical and optical properties of dental ceramics.

## Figures and Tables

**Table 1:** Characteristics of the ceramic systems investigated.

Materials		Manufacturer	Shade	Batch No.	Sintering
**Zirconia**					
3Y-TZP	Lava Plus	3M ESPE, USA	White	669344	1500 °C for 2 h
	Luxen Zr	DENTALMAX, Korea	S2 (A2)	160523-S2-2	
5Y-PSZ	Lava Esthetic	3M ESPE, USA	A2	4638594	
	BruxZir Anterior	Glidewell, USA	250 (A2)	BZ0019752	
	Luxen Smile	DENTALMAX, Korea	SMS2 (A2)	190222-10SMS2-1	
**Glass-Ceramic**					
e.max CAD	High translucent (HT)	Ivoclar Vivadent, Liechtenstein	A2	T02466 V35858 X17428	820 °C for 2 min + 840 °C for 7 min
	Low translucent (LT)		A2	U36939	

**Table 2:** The mass percent composition of each specimen determined by X-ray fluorescence (XRF).

Materials		Component (wt.%)
**Zirconia**		
3Y-TZP	LP	Al (0.0651), Y (5.5603), Zr (91.6982), Hf (2.6765)
	S2	Al (0.0516), Fe (0.0535), Y (5.4426), Zr (91.6896), Hf (2.7627)
5Y-PSZ	LET	Al (0.0346), Y (9.1951), Zr (88.8130), Er (0.1741), Hf (1.7832)
	LEB	Al (0.0391), Y (8.8127), Zr (88.8855), Er (0.4899), Hf (1.7727)
	BA	Ca (0.0217), Y (9.7067), Zr (87.5350), Hf (2.7367)
	SMS2	Al (0.0226), Fe (0.0554), Y (7.8563), Zr (88.3084), Er (1.0339), Hf (2.7235)
**Glass-Ceramic**		
e.max CAD	HT	Al (1.2961), Si (59.7200), P (1.9325), S (0.0211), Li (13.6079), Ca (0.1034), V (0.4735), Zn (3.9805), Sr (0.0604), Zr (3.9142), Ce (5.7366), Tb (2.6394), K (6.5144)
	LT	Al (2.7798), Si (62.7669), P (2.1251), S (0.1007), Li (13.7656), Ca (0.1091), V (0.4351), Zr (2.0843), Ce (7.6236), Tb (2.3141), K (5.8957)

**Table 3:** Physical and mechanical properties of the specimens investigated. Standard deviations in parentheses.

Materials		Density (g/cm^3^)	Modulus (GPa)	Hardness (GPa)	Strength (MPa)	Toughness (MPa·m^1/2^)
**Zirconia**						
3Y-TZP	LP	6.070 ^a^	231	14.09 (0.47)	982.5 (47.1)	5.18 (0.79)
	S2	6.096 ^a^	208	12.74 (0.05)	1054.4 (68.1)	4.34 (0.09)
5Y-PSZ	LE	6.044 ^a^	233	15.47 (0.39)	691.3 (65.4)	3.58 (0.75)
	BA	5.819	221	12.51 (0.12)	538.7 (48.1)	3.34 (0.17)
	SMS2	6.100 ^a^	214	13.16 (0.15)	801.7 (64.5)	3.18 (0.13)
**Glass-ceramic**						
e.max CAD	HT	2.502 ^b^	102 ^a^	5.72 (0.08)	288.5 (31.0) ^a^	2.34 (0.32)
	LT	2.423 ^b^	102 ^a^	6.89 (0.33)	290.1 (27.9) ^a^	2.12 (0.29)

Means with the same superscript letter in each column are not significantly different from each other based on Tukey’s multiple comparison test (*p* > 0.05).
